# Coping tendencies play partial mediating role between social support and anxiety/depression among Chinese keloid patients

**DOI:** 10.3389/fpsyt.2025.1543484

**Published:** 2025-10-15

**Authors:** Yuting Huang, Shu Xu, Yuqi Wu, Zhifeng Gu, Chen Dong, Li Zhang, Xiaodong Chen

**Affiliations:** ^1^ Department of Dermatology, Department of Nursing, Affiliated Hospital of Nantong University, Medical School of Nantong University, Nantong, China; ^2^ School of Nursing and Rehabilitation, Nantong University, Affiliated Hospital of Nantong University, Nantong, China; ^3^ Department of Rheumatology, Research Center of Immunology, Affiliated Hospital of Nantong University, Medical School of Nantong University, Nantong, China; ^4^ Research Center of Clinical Medicine, Affiliated Hospital of Nantong University, Nantong, China

**Keywords:** keloid, anxiety, depression, coping tendencies, social support

## Abstract

**Background:**

In addition to producing pain and itching, keloids can cause psychological symptoms, including anxiety and depression. We aimed to investigate the prevalence and underlying factors of anxiety/depression in Chinese keloid patients and to explore the mediating role between social support and anxiety/depression.

**Methods:**

A total of 202 self-reported questionnaires were collected from keloid patients, including the General Information Questionnaire, Vancouver Scar Assessment Scale (VSS), Patient and Observer Scar Assessment Scale (POSAS), Dermatological Problems Quality of Life Inventory (DLQI), Social Support Rating Scale (SSRS), Simple Coping Styles Scale (SCSQ), Self-Efficacy Scale (GSES) and the Hospital Anxiety and Depression Scale (HADS).

**Results:**

The mean scores for anxiety and depression were 9.82 ± 2.20 and 7.96 ± 2.70, respectively. Annual income (OR = 0.258), GSES score (OR = 0.2955), pain symptoms (OR = 1.281) and coping styles (OR:3.321) were significantly associated with HADS anxiety in keloid patients (P<0.05), whereas use of support (OR = 0.607) was significantly associated with HADS depression in keloid patients (p<0.05). The area under the curve (AUC) for the combined anxiety ROC for annual income, coping styles, pain symptoms and GSES scores was 0.835. PROCESS analysis concluded that coping tendencies partially mediated the relationship between social support and depression, with the mediating effect accounting for 38.58% of the total effect.

**Conclusion:**

Coping tendency in keloid patients played a partial mediating role between social support and depression. Future studies should further explore how training in coping tendencies can enhance the effectiveness of social support to more effectively prevent and reduce depressive symptoms.

## 1 Introduction

Keloids are fibroproliferative disorders that predispose patients with abnormally healing wounds from trauma, surgery, blisters, inflammation, acne, or vaccinations (1, 2). Keloids usually cause discomfort such as itching and pain, and in addition, patients tend to suffer from psychological distress due to negative body image, which can seriously affect quality of life (3, 4). Compared to the general population, patients with facial scars have a considerable burden of anxiety and depression. Excessive preoccupation with body image in turn increases negative mental health conditions, leading to a reduced quality of life (5).

Anxiety and depression are associated with a variety of conditions such as primary Sjögren’s syndrome, acne, alopecia, and psoriasis (6–9). Moderate-to-severe atopic dermatitis was found to be significantly associated with a marked deterioration in mental health in a study of anxiety and depressive states in American adults with atopic dermatitis (10). However, assessment of mental health status is rarely included in clinical practice. Some studies have shown that psychological stress has an impact on the recurrence of postoperative keloids (11). Therefore, exploring the role of anxiety/depression in the development and prognosis of keloids is important and may be an effective target for intervention.

Francieli Sufredini et al. demonstrated that providing good social support to perinatal women can reduce the risk of anxiety and depression (12). Xiaoying Ning et al. proposed in their study on the psychological status of vitiligo patients in China that low social support constitutes one of the risk factors for depression among vitiligo patients (13). Social support networks may not only reduce anxiety and depression symptoms directly, but also promote mental health in indirect ways such as enhancing one’s coping strategies and increasing self-efficacy. Understanding and strengthening these aspects of support is therefore crucial to the prevention and treatment of anxiety and depression.

Negative coping styles, such as avoidance and self-blame, may exacerbate an individual’s psychological stress. Previous research suggests that negative coping styles may increase the risk of anxiety (14–16). Coping with the effects of keloids may be a difficult lifelong process. The role of coping tendencies in linking anxiety/depression in Chinese keloid patients is not yet understood, so this study attempts to fill this gap.

Previous reports in the literature on other diseases suggest that anxiety and depression in patients with malignant melanoma, burns, and lupus are influenced by a variety of factors such as the patient’s socio-economic status, disease state, social support, and coping styles (17–19), as well as adversely affecting the patient’s quality of life (20, 21). Keloid can exacerbate anxiety/depression due to the visible scarring and physical discomfort; however, there is a lack of such studies in keloid patients to reveal the prevalence of anxiety and depression in keloid patients, the factors influencing it, and the central role played by social support and coping styles, especially in the Chinese population. Therefore, this study focuses on keloids as a driver of psychological distress in order to investigate (1): to investigate the prevalence of anxiety/depression in keloid patients (2) to explore the factors and possible risk factors associated with anxiety/depression; and (3) to explore the relationship between coping styles, social support, and anxiety/depression in keloid patients.

## 2 Methods

### 2.1 Participants

The study was carried out in the Department of Dermatology, Affiliated Hospital of Nantong University from October 2022 to January 2024. The 202 keloid patients were from the outpatient or inpatient wards while 202 healthy controls were recruited from individuals undergoing routine health checkups, with no history of dermatologic diseases. All of keloid patients met the diagnostic criteria for keloids. Exclusion criteria for patients with keloids included (1) Patients with metabolic disorders (e.g., diabetes, cardiovascular disease) or systemic conditions linked to affective disorders were excluded to minimize confounding effects on psychological outcomes (2); pregnant or lactating women; and (3) incomplete clinical case information. This cross-sectional study was approved by the Ethics Committee of Nantong University Hospital (2021-K132-01). All participants were informed about the study and signed an informed consent form.

### 2.2 Demographic and clinical characteristics

Demographic variables included age, gender, marital status, education, annual per capita income, place of residence, and lifestyle. Clinical variables included family history, duration of disease, comorbidities, onset triggers, self-perceived symptoms, skin lesion manifestations, past treatment outcomes, clinic visits, and scar assessments: Vancouver Scar Scale (VSS) and Patient and Observer Scar Assessment Scale (POSAS). Patient-reported outcomes were evaluated using several standardized measures: the Dermatologic Life Quality Index (DLQI) for skin-related quality of life, the Social Support Rating Scale (SSRS) for perceived social support, the Simple Coping Style Questionnaire (SCSQ) for coping strategies, the General Self-Efficacy Scale (GSES) for self-confidence in managing challenges, and the Hospital Anxiety and Depression Scale (HADS) for psychological distress.

### 2.3 Statistical analysis

SPSS 26.0 software was used to analyze the data. Normal distribution was expressed as mean (± standard deviation) and non-normal distribution was expressed as median (25th and 75th percentile) or number (percentage). Categorical variables were expressed as values and percentages. Independent t-test and chi-square test were used to evaluate the differences between continuous and categorical variables in keloid patients. The PROCESS macro model 4 developed by Hayes was used to test the mediating effect using bootstrap method (22). The resamples were 5000 and the confidence interval was 95%. A statistically significant mediation effect was indicated if the confidence interval did not contain 0. A difference of P<0.05 was considered statistically significant.

## 3 Results

### 3.1 Patient characteristics

The demographic characteristics of the study participants are shown in **Table 1**. The mean age did not differ significantly among healthy controls, and keloid patients (31.61 ± 6.00, and 32.87 ± 11.21 years, respectively; P>0.05). However, the gender distribution varied significantly across groups, the healthy control group consisted predominantly of females (90.1%), whereas the keloid group included approximately equal proportions of males (50.5%) and females (49.5%)(P < 0.001).Compared with healthy controls, keloid patients had significantly higher rates of anxiety (13.4%) and depression (10.4%). This supports the conclusion that keloid scarring is associated with increased psychological burden.

**Table 1:** The anxiety/depression rate of health people and keloid patients.

Variable	General (n=404)	Health controls(n=202)	Keloid patients (n=202)	*P*
Age^a^	32.24 ± 9.00	31.61 ± 6.00	32.87 ± 11.21	0.159
Gender^b^				<0.001**
Male (0)	122(30.2%)	20(9.9%)	102(50.5%)	
Female (1)	282(69.8%)	182(90.1%)	100(49.5%)	
HADS-anxiety, yes^b^	34(8.4%)	7(3.5%)	27(13.4%)	<0.001**
HADS-depression, yes^b^	30(7.4%)	9(4.5%)	21(10.4%)	0.023*

a Values are presented as the Mean ± SD and analyzed by independent samples t-test.

^b^Values are presented as the number (%) analyzed by chi-square tests.

*P < 0.05, **P < 0.001.

### 3.2 Social Economics, Psychological, and clinical characteristics of keloid patients

A total of 202 patients with keloids were investigated in this study. The general demographic and lifestyle analysis is shown in **Table 2**. The analysis of the results showed that the mean age of keloid patients was 31.39 ± 11.53 years (P>0.05), and the annual household income was less than 50,000 yuan per year in 45 (22.3%) and more than 100,000 yuan in 56 (55.4%)(P < 0.05). When comparing keloid patients with anxiety/depression to those without, no statistically significant differences were observed in gender, place of residence, marital status, education level, or lifestyle behavior (P>0.05). 117 patients (57.9%) had itching and pain at the keloid site and 58 patients (28.7%) had infection at the lesion site. There was no statistically significant difference in terms of duration of disease, family history, lesion site, color, blood supply and thickness (P>0.05). Additional information is shown in **Table 3**.

**Table 2:** Differences between demographic of anxiety/depression and non-anxiety/depression patients.

Characteristics	Number of cases	Non-anxiety	Anxiety	*P*	Non-depression	Depression	*P*
Age, years^a^	31.39 ± 11.53	31.05 ± 10.93	33.59 ± 14.95	0.287	31.60 ± 11.50	29.57 ± 11.96	0.468
Gender^b^				0.793			0.117
Male	102(50.5)	89(50.9)	13(48.1)		88(48.6)	14(66.7)	
Female	100(49.5)	86(49.1)	14(51.9)		93(51.4)	7(33.3)	
Residency^b^				0.080			0.353
Urban	160(79.2)	140(80.0)	20(74.1)		145(80.1)	15(71.4)	
Village	42(20.8)	35(20.0)	7(25.9)		36(19.9)	6(28.6)	
Marital status^b^				0.369			0.361
Married	96(47.5)	81(46.3)	15(55.6)		88(48.6)	8(38.1)	
Other	106(52.5)	94(53.7)	12(44.4)		93(51.4)	13(61.9)	
Educational level^b^				0.686			0.446
< 18 years	90(44.6)	77(44.0)	13(48.1)		79(43.6)	11(52.4)	
≥ 18ars	112(55.4)	98(56.0)	14(51.9)		102(56.4)	10(47.6)	
Year per capita income, yuan^b^				0.005*			0.377
< 50,000	45(22.3)	33(18.9)	12(44.4)		38(21.0)	7(33.3)	
50,000-100,000	101(50.0)	94(53.7)	7(25.9)		91(50.3)	10(47.6)	
>100,000	56(27.7)	48(27.4)	8(29.6)		52(28.7)	4(19.0)	
Tobacco use^b^				0.329			0.020*
Yes	32(15.8)	26(14.9)	6(22.2)		25(13.8)	7(33.3)	
No	170(84.2)	149(85.1)	21(77.8)		156(86.2)	14(66.7)	
Alcohol use^b^				0.825			0.542
Yes	13(6.4)	11(6.3)	2(7.4)		11(6.1)	2(9.5)	
No	189(93.6)	164(93.7)	25(92.6)		170(93.9)	19(90.5)	
No-light diet^b^				0.690			0.602
Yes	134(66.3)	117(66.9)	17(63.0)		119(65.7)	15(71.4)	
No	68(33.7)	58(33.1)	10(37.0)		62(34.3)	6(28.6)	

^a^t-tests; ^b^χ2 analyses. *P < 0.05.

**Table 3:** Differences between clinical characteristics of anxiety/depression and non-anxiety/depression patients.

Clinical characteristics	Number of cases	Non-anxiety	Anxiety	*P*	Non-depression	Depression	*P*
Duration^b^				0.680			0.530
≤ 5 years	95(47.0)	82(47.4)	13(48.1)		96(53.6)	9(42.9)	
≥ 10 years	105(52.0)	47(27.2)	7(25.9)		50(27.9)	4(19.0)	
Age of initial onset^b^				0.335			0.707
≤ 18 years	67(33.2)	57(32.8)	10(37.0)		58(32.2)	9(42.9)	
Family history,yes^b^	42(20.8)	35(20.0)	7(25.9)	0.480	39(21.5)	3(14.3)	0.438
Causes Infection,yes^b^	121(59.9)	101(57.7)	20(74.1)	0.568	107(59.1)	14(66.7)	0.504
Symptoms^b^				0.008*			0.120
Itching	52(25.7)	51(29.1)	1(3.7)		51(28.2)	1(4.8)	
Pain	2(1.0)	1(3.7)	1(3.7)		2(1.1)	0(0.0)	
Itching with pain	117(57.9)	94(53.7)	23(85.2)		100(55.2)	17(81.0)	
Lesions with infection, yes^b^	58(28.7)	44(25.1)	14(51.9)	0.004*	48(26.5)	10(47.6)	0.043*
Lesions at exposed sites, yes^b^	67(33.2)	59(33.7)	8(29.6)	0.675	60(33.1)	7(33.3)	0.986
Pigmentation, hyperpigmentation, yes^b^	93(46.0)	96(54.9)	16(59.3)	0.668	83(45.9)	10(47.6)	0.878
Vascularity, red or purple, yes^b^	87(43.1)	76(43.4)	11(40.7)	0.793	78(43.1)	9(42.9)	0.983
Pliability,firm, ropes or contracture^b^	179(88.6)	154(88.0)	25(92.6)	0.484	161(89.0)	18(85.7)	0.659
Height, ≥2mm^b^	107(53.0)	96(54.9)	11(40.7)	0.171	97(53.6)	10(47.6)	0.604
Past treatment effects^b^				0.507			0.228
Ineffective	119(58.9)	103(58.9)	16(59.3)		83(65.9)	10(90.9)	
Effective	19(9.4)	18(10.3)	1(3.7)		37(29.4)	1(9.1)	
VSS^a^		13.85 ± 2.39	13.78 ± 2.21	0.874	13.85 ± 2.38	13.76 ± 2.21	0.864
PSAS^a^		47.41 ± 11.10	51.63 ± 13.31	0.127	47.92 ± 11.21	48.43 ± 13.83	0.872
OSAS^a^		36.35 ± 9.95	41.48 ± 9.86	0.017*	36.77 ± 10.21	39.33 ± 8.63	0.217
POSAS^a^		83.75 ± 16.69	93.11 ± 18.67	0.020*	84.69 ± 17.11	87.76 ± 18.29	0.470
DLQIb		7.17 ± 4.43	13.52 ± 6.09	<0.001**	7.51 ± 4.71	12.38 ± 6.64	0.003*
Moderate impact and above	129(63.9)	105(60.0)	24(88.9)		112(61.9)	17(81.0)	
Social support^a^		34.00 ± 6.66	30.48 ± 7.23	0.023*	34.09 ± 6.59	28.67 ± 7.03	0.003*
Objective support		8.45 ± 2.96	7.59 ± 3.08	0.187	8.51 ± 2.93	6.81 ± 3.06	0.023*
Subjective suport		18.30 ± 3.63	16.59 ± 3.72	0.033*	18.30 ± 3.60	16.10 ± 3.83	0.019*
Utilization of support		7.25 ± 1.96	6.30 ± 1.44	0.004*	7.28 ± 1.90	5.76 ± 1.61	<0.001**
Coping tendenciesb							
Positive	123(60.9)	114(65.1)	9(33.3)	<0.001**	119(65.7)	4(19.0)	<0.001**
Negative	79(39.1)	61(34.9)	18(66.7)	0.388	62(34.3)	17(81.0)	0.795
Anxiety, yes^a^	27(13.4)	2.82 ± 2.29	9.82 ± 2.20	<0.001**			
Depression, yes^a^	21(10.4)				2.38 ± 2.55	7.96 ± 2.70	<0.001**
GSES^a^		2.51 ± 0.56	2.21 ± 0.48	0.006*	2.49 ± 0.56	2.27 ± 0.47	0.059

^a^t-tests; ^b^χ2 analyses.

*P < 0.05, **P < 0.001.

### 3.3 Differences between anxiety/depression and non-anxiety/depression in keloid patients

Comparison of clinical and psychological variables between anxious/depressed and non-anxious/depressed keloid patients is shown in **Table 3**. The number of anxious patients with accompanying pain and or itching was 25, and the number of lesions with infections was 14 (51.9%), and all these disease characteristics showed statistical significance ((P<0.05). Compared to non-anxious patients, anxious keloid patients had OSAS score (41.48 ± 9.86), POSAS score (93.11 ± 18.67), DLQI score (13.52 ± 6.09), social support score (30.48 ± 7.23) (including objective support (7. 59 ± 3.08), subjective support (16.59± 3.72), support utilization (6.30 ± 1.44)) and GSES score (2.21 ± 0.48) were lower, and the proportion of positive coping was even lower (P<0.05). Among them, 129 patients (63.9%) had moderate or higher DLQI score results.

Depressed patients with keloids had 10 with concomitant infection at the skin lesion site (P<0.05), higher DLQI score (12.38 ± 6.64) and lower social support score (28.67 ± 7.03) including objective support (6.81 ± 3.06), subjective support (16.10 ± 3.83) and support utilization (5.76 ± 1.61). Compared with keloids patients without depression, the results of DLQI scores of 112 patients (61.9%) were moderately affected or more serious (P < 0.05), and the difference between VSS scores and POSAS scores was not statistically significant (P>0.05).

### 3.4 Logistic regression analysis for the HADS-anxiety and depression

In the anxiety population of keloid patients, binary logistic regression analysis showed that annual income >100,000 (OR = 0.258; P<0.05), GSES score (OR = 0.295; P<0.05), pain symptoms (OR = 1.281; P<0.05), and coping tendency (OR: 3.321; P<0.05) were significantly associated with keloid patients’ HADS-anxiety. This suggests that annual income, GSES score, pain symptoms and coping tendency are predictors of anxiety in keloid patients (**Table 4**). On the basis of this analysis, the subjects’ work characteristic curves (ROC) were plotted to assess the predictive value of the above factors for anxiety and depression, respectively, with an AUC value of 0.835 for anxious patients and 0.792 for depressed patients.

**Table 4:** Result of forward stepwise ordered logit regression models in anxiety and depression.

Item	Variables	Beta	SE	P-value	Exp(b)	95%CI
Anxiety	Income,< 50,000 yuan	0.353	0.610	0.562	1.424	(0.431,4.706)
	Income, >100,000 yuan	1.354	0.61	0.028	0.258	(0.077,0.863)
	GSES	1.222	0.113	0.013	0.295	(0.112,0.775)
	Keloid with pain	0.247	0.094	0.009	1.281	(1.065,1.540)
	Lesions in abdomen	1.183	0.614	0.054	3.263	(0.979,10.869)
	Coping tendencies	1.200	0.489	0.014	3.321	(1.273,8.664)
Depression	Coping tendencies, negative	2.09	0.577	0.000	8.157	(2.631,25.293)

CI, confidence interval; GSES, general self-efficacy scale, R^2^ = 0.314.

In the depressed population of keloid patients, negative coping tendency (OR: 3.321; P<0.05) was significantly associated with HADS depression in keloid patients, indicating that negative coping tendency was a predictor of depression in keloid patients (**Table 3**).

### 3.5 Mediation effects of the coping tendencies

We used PROCESS to analyze the mediating effects between social support, anxiety/depression and coping tendencies. The results are shown in **Table 5**. When coping tendency was tested as the outcome variable, the R value was 0.4836, P<0.01, which was statistically significant, and the regression coefficient a = 0.5440, P<0.001, 95% CI (0.4067, 0.6812), suggesting that the independent variable, social support, had a positive effect on the mediating variable, coping tendency.

**Table 5:** Mediating effects of coping styles between social support and anxiety/depression.

Item	Predictor variables	Outcome variables	R	F	β	T	95%CI
Anxiety	Social support	Anxiety	0.2179	9.9704**	-0.1053	-3.1576**	(-0.1711,-0.0395)
	Social support	Coping tendencies	0.4836	61.0610***	0.5440	7.8142***	(0.4067,0.6812)
	Social support				-0.0678	-1.7928**	(-0.1423,0.0068)
		Anxiety	0.2593	7.1737**			
	Coping tendencies				-0.0690	-2.0534**	(-0.1353,-0.0027)
Depression	Social support	Depression	0.3452	27.0581***	8.5384	-5.2017***	(-0.2227,-0.1003)
	Social support	Coping tendencies	0.4836	61.0610***	0.5440	7.8142***	(0.4067,0.6812)
	Social support				-0.0992	-2.8868**	(-0.1670,-0.0314)
		Depression	0.4210	21.4315***			
	Coping tendencies				-0.1145	-3.7471***	(-0.1747,-0.0542)

Variables in the model are normalized, the same as below.

*p<0.05, **p<0.01, ***p<0.001.

**Figure 1:**
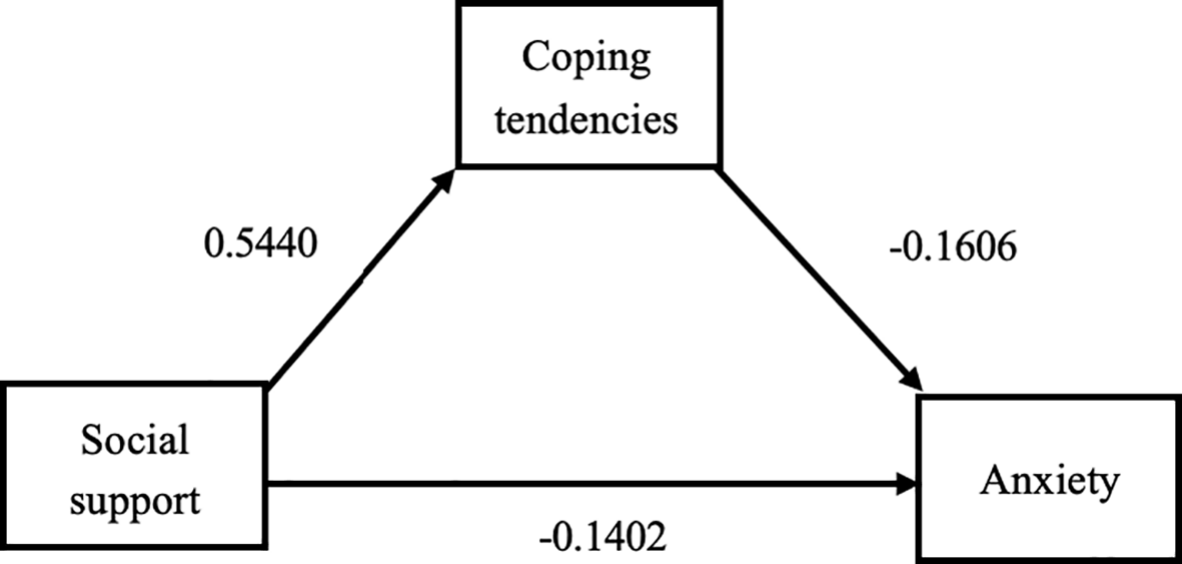
Path diagram of the mediating effect of coping styles between social support and anxiety.

When anxiety was tested as an outcome variable, the R-value was 0.2593, P<0.01, indicating that the model was statistically significant. The local regression coefficient b (effect of coping style on anxiety) = -0.0690, significant (P<0.01), 95% CI (-0.1353, -0.0027). Local regression coefficient c’ (direct effect of social support on anxiety) = -0.0678, significant (P< 0.01), 95% CI (-0.1423, 0.0068). Therefore, it can be concluded that coping tendency has a mediating and partially mediating effect (**Figure 1**).

**Figure 2:**
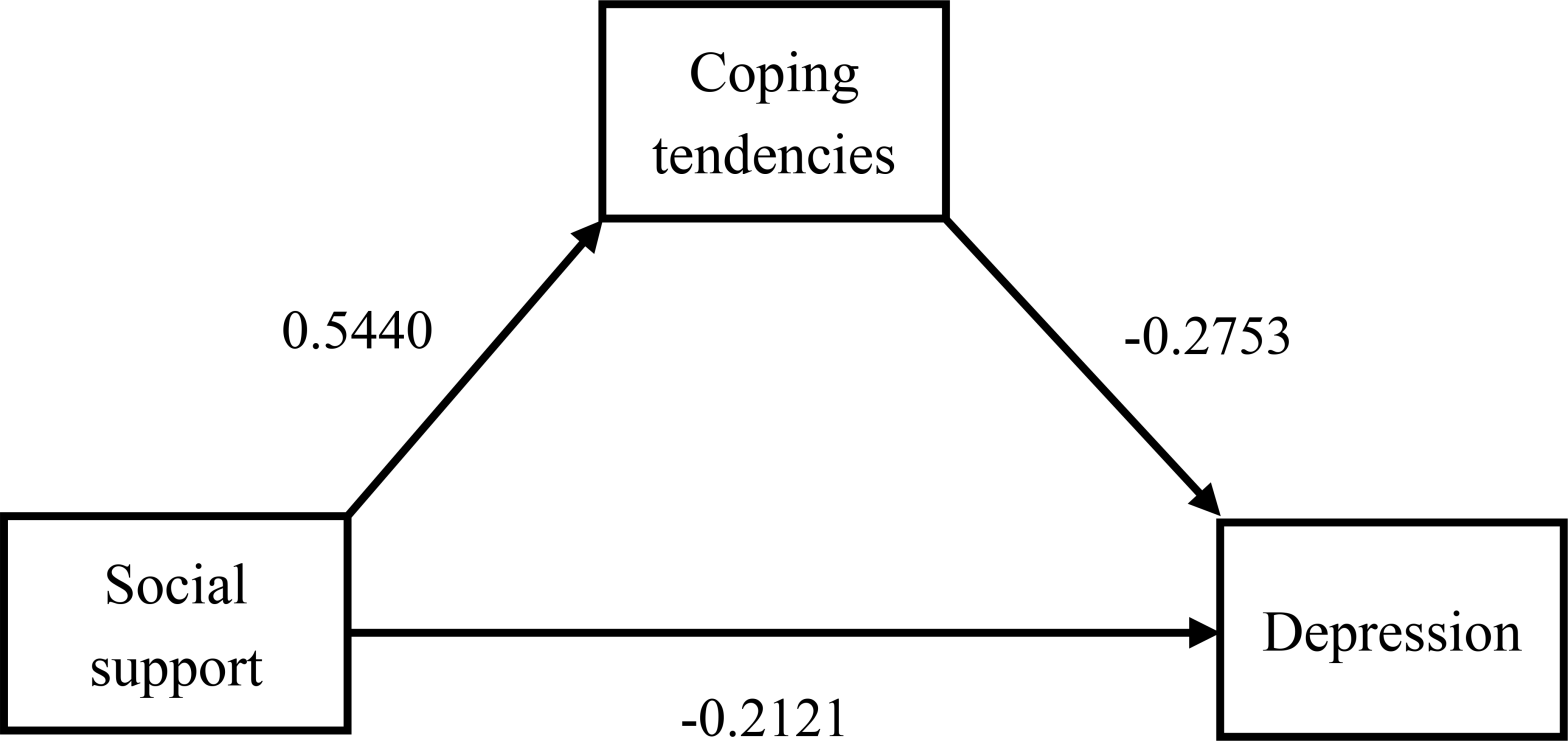
Path diagram of the mediating effect of coping styles between social support and depression.

When depression was tested as an outcome variable, the R value was 0.4210, P<0.001, indicating that the model was statistically significant. The local regression coefficient b (effect of coping style on depression) = -0.1145, significant (P< 0.001), 95% CI (-0.1747, -0.0542). Local regression coefficient c’ (direct effect of social support on depression) = -0.0992, significant (P>0.01), 95% CI (-0.1670, -0.0314). Therefore, it can be concluded that coping tendency has a mediating and partially mediating effect (**Figure 2**).

We validated the mediating effect using the bias-corrected percentile Bootstrap method (5000 replicate extractions). The results are shown in **Table 6**, where the total effect value for anxious patients was -0.1053 and the mediated effect value was 35.61%. 95% CI (-0.0725,-0.0025) excluding 0, which again validates the presence of an indirect effect. Similarly, the total effect value for depressed patients was -0.1615 with a mediated effect value of 38.58%. 95% CI (-0.0968,-0.0296) excluding 0, which again validates the presence of an indirect effect. Therefore, we conclude that the mediation effect in this model is statistically significant and that the coping tendency is partially mediated.

**Table 6:** Bootstrap tests for mediating effects of coping tendencies, social support, and anxiety/depression.

Item	Variables	Effect	BootSE	BootLLCI	BootULCI
Anxiety	Total effect	-0.1053	0.0334	-0.1711	-0.0395
	Direct effect	-0.0678	0.0378	-0.1423	0.0068
	Indirect effect	-0.0375	0.0175	-0.0725	-0.0025
Depression	Total effect	-0.1615	0.0310	-0.2227	-0.1003
	Direct effect	-0.0992	0.0344	-0.1670	-0.0314
	Indirect effect	-0.0623	0.0173	-0.0968	-0.0296

## 4 Discussion

To the best of our knowledge, this study was the first to investigate the prevalence of anxiety and depression in Chinese keloid patients, which were 13.4% and 10.4%, respectively. Self-perceived symptoms such as itching and pain, infection, social support, quality of life, and self-efficacy are all factors that may be related to the anxiety and depression status of keloid patients. Meanwhile, annual income, GSES score, pain symptoms and coping tendency were predictors of anxiety and negative coping tendency was a predictor of depression in keloid patients. In addition, we explored for the first time the indirect role of negative coping tendency as a mediator, which is important for early prevention and intervention of anxiety/depression in keloid patients. In this study, we found for the first time that the occurrence of anxiety and depression in keloid patients had an extremely negative impact on the quality of life of patients, suggesting that it is particularly important to control the psychological state of patients.

We found that low-income keloid patients often lack necessary medical information and psychological support, which, together with possible concomitant bad habits such as smoking, are more likely to increase the incidence of malignant mental health conditions. Therefore, increased attention to low-income groups, optimal allocation of medical resources, and universal health education are important for preventing anxiety and depression conditions in keloid patients.

Keloids can be considered as one of the immunocompromised cutaneous areas (ICD), which means that keloid areas are more susceptible to infections (23). In addition, immune cells, such as macrophages and T-lymphocytes, in keloids constantly produce inflammatory factors, which exacerbate the inflammatory response and lead to more pronounced itching and pain (24, 25). We found that itching and pain were among the self-sensed symptoms caused by the skin lesions which dominate and severely affect the quality of life of patients, which is consistent with the results of previous studies. Moreover, we found that anxiety and depression states were influenced by subjective feelings such as pain and itching. Therefore, extra attention needs to be paid to the mental health of patients with keloid site infection and itchy pain symptoms in clinical practice.

Within the keloid patient group, anxious patients showed higher scar severity, lower quality of life, less social support, lower self-efficacy, and greater difficulty in adopting positive coping strategies compared with non-anxious patients. Quality of life, social support and ability to cope positively were more negatively affected in depressed keloid scar patients than in non-depressed patients. Although these results are expected, they are noteworthy in the context of keloid disease, because psychological symptoms in dermatological patients are not identical to those seen in individuals with primary psychiatric diagnoses of anxiety or depression. The impact of visible skin lesions, persistent itching and pain, and body image concerns may contribute to unique pathways through which anxiety and depression impair quality of life in keloid patients.

Social support is significantly negatively correlated with anxiety and depression, and anxious and depressed keloid patients receive less social support in terms of emotional support, companionship and guidance. Keloids are difficult to treat and prone to recurrence, and the treatment process is not only expensive, but also the lack of understanding or misunderstanding by family members, friends, or other groups, or even the failure to provide any material help or action support, may aggravate the patient’s psychological burden (26, 27). Therefore, it is important for families and society to provide more care. This study also reveals the important role of coping styles for keloid patients. Previous research has demonstrated a negative association between acculturative stress and positive coping (28). In China, collectivist cultural norms place a strong emphasis on family support, yet may simultaneously contribute to the stigmatization of visible scars. Moreover, in societies with stringent beauty standards, the acceptance of scars can be particularly challenging. Future research should investigate culturally tailored interventions, such as community-based storytelling to normalize the experience of keloid scarring, or the integration of traditional treatments with psychosocial support. Keloid patients are more likely to use negative coping styles, which further aggravates the increased incidence of anxiety and depression. Therefore, we need to provide psychological support to keloid patients in a variety of ways to reduce their psychological stress, improve their quality of life, and reduce anxiety and depression symptoms. Targeted interventions should integrate coping skills training, such as cognitive-behavioral therapy (CBT) modules, to help individuals reframe negative coping mechanisms (e.g., avoidance) into adaptive strategies (e.g., problem-solving). Additionally, social support enhancement through structured peer-support groups or family education programs can help reduce stigma and improve emotional and instrumental support. Finally, a multidisciplinary care approach, including collaborative dermatology-psychiatry clinics, ensures that both physical and psychological symptoms are addressed simultaneously for comprehensive treatment.

Unique to the present study is the novel finding that coping tendencies partially mediate the effect between social support and depressive symptoms. First, we highlight that patients with poorer social support are more likely to experience anxiety/depressive symptoms, and consistent with previous research, social support is an important protective factor associated with lower levels of anxiety/depression. This highlights the importance of strengthening social connections and community support networks in preventing and reducing depressive symptoms. More importantly, our data suggest that coping styles are an important way in which social support influences depression, which has not been fully explored in previous research. Thus, when individuals perceive higher levels of social support, they are more likely to adopt positive coping styles in response to stress, thereby reducing or avoiding depressive symptoms. Furthermore, these findings suggest that improving individuals’ coping skills may be an effective way to enhance the effects of social support. Unless individuals are able to use this support effectively through positive coping strategies, the provision of social support alone may not be sufficient to prevent or alleviate depression. Therefore, future interventions and treatment plans should take into account the need to strengthen an individual’s social support network and coping skills, especially when facing life challenges.

The study’s single-center design and homogeneous Chinese cohort may limit generalizability to other populations with varying cultural, economic, or healthcare contexts. Multicenter studies with diverse demographics are needed to validate these findings in the future. Self-reported measures (e.g., HADS, SSRS) may be influenced by recall bias or social desirability. Future work could combine subjective scales with clinician-administered assessments or biomarkers (e.g., cortisol levels) to triangulate data.

## 5 Conclusion

In summary, this study reveals the disease characteristics of keloid scars, suggests possible predictors of anxiety and depressive symptoms in keloid patients, and creatively analyzes the partial mediating role of coping tendencies among keloid patients between social support and depression. These findings provide an important theoretical basis and practical guidelines for developing more integrated and effective mental health intervention strategies. Future research should further explore how training in positive coping strategies can increase the effectiveness of social support to more effectively prevent and alleviate depressive symptoms and ultimately improve patient prognosis.

## Data Availability

The raw data supporting the conclusions of this article will be made available by the authors, without undue reservation.
